# Acute Transverse Myelitis and Cervical Spondylodiscitis Associated With Staphylococcus aureus Bacteremia: A Case Report

**DOI:** 10.7759/cureus.116447

**Published:** 2026-09-17

**Authors:** Maria Lypiridou, Konstantinos Kalafatakis, Kyriaki Astara, Anna Margoni, Georgios Stouraitis

**Affiliations:** 1 Department of Neurology, 417 Army Share Fund Hospital, Athens, GRC; 2 Department of Neurology, Pammakaristos Hospital, Athens, GRC

**Keywords:** cervical spine, discitis, osteomyelitis, spondylitis, spondylodiscitis, staphylococcus aureus, transverse myelitis

## Abstract

Acute transverse myelitis (ATM) is a rare, acquired neuroimmune disorder of the spinal cord that manifests with acute-onset weakness, sensory alterations, and autonomic dysfunction. We report a case of a 56-year-old immunocompetent female patient who presented with fever, flaccid quadriparesis, nuchal rigidity, and markedly elevated inflammatory biomarkers. Blood cultures came back positive for methicillin-sensitive *Staphylococcus aureus*, and magnetic resonance imaging (MRI) of the cervical spine demonstrated increased T2 signal between C4 and C7, inflammation of the anterior perivertebral soft tissues between C5 and C7, and pathological tissue in the epidural space between C3 and C7. Additional extensive laboratory and imaging workup was performed to obtain a prompt and accurate diagnostic conclusion, while considering the broad differential diagnosis. Experience gained from this report is important to ensure timely diagnosis and optimal management of this rare clinical entity, as the most significant prognostic factor for a favorable outcome is early diagnosis and appropriate pharmacological treatment.

## Introduction

Acute transverse myelitis (ATM) is a rare, focal neuroinflammatory disorder of the spinal cord, affecting one or more levels, with an estimated annual incidence ranging between 1.3 and 4.6 cases per million and a mean age of occurrence between 35 and 40 years [1]. ATM includes a pathobiologically heterogeneous syndrome characterized by acute or subacute spinal cord dysfunction, resulting in varying degrees of muscle weakness, and sensory and autonomic dysfunction (bladder, bowel, and sexual) below the level of the lesion [2]. The diagnosis may incorporate the term idiopathic (a significant portion of the total cases) when no features suggestive of another cause are identified, despite a thorough clinical, imaging, and laboratory investigation, or disease-associated when related to another disease process [3]. Etiologies for disease-associated ATM can be broadly classified as infectious, parainfectious, due to systemic autoimmune disorders (vasculitis, mixed connective tissue diseases, and collagen vascular disease), paraneoplastic, or demyelinating (multiple sclerosis and neuromyelitis optica spectrum disorders). Other conditions that may mimic the clinical and radiological appearance of ATM include non-inflammatory etiologies, such as spinal cord infarction, compressive myelopathy, radiation myelitis, trauma, and nutritional deficiencies [3].

Bacterial involvement of the spinal cord parenchyma is considerably rarer than viral or parainfectious myelitis, and the mechanisms by which bacteria produce cord injury are heterogeneous [1]. Direct intramedullary invasion may occur through hematogenous seeding during bacteremia or by contiguous extension from an adjacent focus such as vertebral osteomyelitis, a paravertebral collection, or an epidural abscess. Alternatively, bacteria adjacent to but not within the cord may cause injury indirectly, through the accumulation of purulent exudate in the subarachnoid space, septic thrombophlebitis and venous congestion, or mechanical compression. A third mechanism is immune-mediated: myelitis frequently follows rather than accompanies the initial infection, a temporal pattern that suggests a parainfectious immunological response rather than direct microbial injury, and molecular mimicry between microbial and neural antigens has been proposed as the underlying process [3]. These mechanisms are not mutually exclusive, and in an individual patient, they are difficult to distinguish because the clinical presentation, cerebrospinal fluid profile, and neuroimaging appearances overlap substantially between them.

*Staphylococcus aureus *(*S. aureus*) is the organism most frequently responsible for native vertebral osteomyelitis and spondylodiscitis, infections of the vertebral body and intervertebral disc, respectively, which most often arise from hematogenous seeding during bacteremia and carry a substantial risk of epidural and paravertebral extension [4]. *Staphylococcus aureus* bacteremia is itself a serious condition with a well-recognized capacity for metastatic seeding to bone, joints, heart valves, and the central nervous system (CNS) [5]. Despite this, myelitis attributable to *S. aureus* is exceptionally uncommon, with only a small number of cases reported. The infrequency of the association creates a specific diagnostic difficulty: when acute transverse myelitis occurs in a patient with staphylococcal bacteremia and adjacent vertebral infection, it is not straightforward to determine whether the cord lesion represents direct infectious involvement, an indirect consequence of the adjacent infection, or a parainfectious immune-mediated process occurring in a patient who also happens to have a staphylococcal infection. The distinction is not merely academic, since it bears on the duration and route of antimicrobial therapy and whether immunomodulatory treatment is appropriate. We present a case in which this question could not be definitively resolved, and we discuss the evidence bearing on each possibility.

## Case presentation

A 56-year-old woman presented to the emergency department with a 10-day history of generalized weakness, fatigue, and progressively deteriorating ability to walk. She also complained of nasal congestion, sore throat, and severe neck pain. The patient reported a history of hypothyroidism, for which she was receiving treatment with levothyroxine 62 μg daily, and vitiligo. She was not a smoker, nor was she using illicit drugs or consuming alcohol, and her family history was unremarkable. Upon admission, monitoring of vital signs revealed a body temperature of 38.2°C (other vitals: blood pressure systolic/diastolic, 142/78 mmHg; heart rate, 78 bpm; and oxygen saturation, 97%). On physical examination, the respiratory system, heart, and bowel sounds were normal. Abdominal palpation was normal as well. Inspection of the oropharynx revealed erythema without tonsillar exudate. On neurological examination, the patient was fully oriented with intact cranial nerve function, but she showed signs suggestive of meningeal irritation (nuchal rigidity), and motor function assessment revealed generalized weakness.

Computed tomography (CT) of the brain, performed on the day of admission, was normal, and CT of the cervical spine showed no acute compressive lesion. A lumbar puncture was subsequently performed on the same day. Blood chemistry showed a marked systemic inflammatory response (Table 1). The cerebrospinal fluid (CSF) demonstrated a lymphocyte-predominant pleocytosis, hypoglycorrhachia with a CSF:serum glucose ratio of 0.40, a mildly elevated lactate dehydrogenase (LDH) concentration, and a markedly elevated total protein concentration (Table 2). This combination was compatible with an infectious process of the CNS but was not sufficiently specific to establish an etiology.

**Table 1:** Blood chemistry at hospital admission CRP: C-reactive protein, GLU: glucose, HCT: hematocrit, HGB: hemoglobin, K: potassium, Na: sodium, NEU%: neutrophils (percentage of total leukocytes), PCT: procalcitonin, WBC: white blood cell count

Readout	Value	Reference normal range
WBC	36.2 K/μL	5-10 K/μL
NEU%	93.9%	40%-60%
HCT	28.4%	36%-48%
HGB	10 g/dL	11.6-15 g/dL
Na	125 mmol/L	135-145 mmol/L
K	3.4 mmol/L	3.5-5.2 mmol/L
GLU	103 mg/dL	70-100 mg/dL
CRP	30.5 mg/dL	<0.3 mg/dL
PCT	1.03 ng/mL	<0.1 ng/mL

**Table 2:** CSF analysis at hospital admission *Serum glucose, measured on a separate sample: 73 mg/dL; CSF: serum glucose ratio 0.40 CSF: cerebrospinal fluid, GLU: glucose, LDH: lactate dehydrogenase, LYMPH: lymphocytes (percentage of total nucleated cells), PRO: protein

Readout	Value	Reference normal range
Cell count	130 cells/μL (88% LYMPH)	0-5 cells/μL
GLU*	29 mg/dL	50-80 mg/dL
LDH	76 U/L	<40 U/L
PRO	885 mg/dL	15-60 mg/dL

The patient was immediately treated empirically for bacterial meningoencephalitis with a combination of intravenous ceftriaxone, vancomycin, ampicillin, and acyclovir while waiting for the results of blood and CSF cultures and other microbiological analyses. During the following hours, the patient deteriorated and progressively developed flaccid quadriparesis and a bilateral sensory dysfunction at the C4 level, all indicating acute cervical spinal cord injury. On formal neurological examination, the patient was alert with preserved higher cortical function, normal oculomotor responses, and intact cranial nerves. Muscle strength (Medical Research Council (MRC) grade) was 4/5 proximally and 3/5 distally in the right upper limb, 5-/5 proximally and 3/5 distally in the left upper limb, and 2+/5 in both lower limbs. Deep tendon reflexes were abolished in all four limbs, plantar responses were absent, and Barré and Mingazzini signs were positive bilaterally; Hoffmann's sign was absent. Tests of cerebellar function were normal, but the patient was unable to maintain a standing position. Bladder and bowel function were preserved throughout.

The patient underwent electrodiagnostic testing, and peripheral nerve or primary myopathic disorders were excluded as the cause of the presentation. Subsequently, on hospital day 10, magnetic resonance imaging (MRI) of the brain and cervical spine was performed on a 1.5 T scanner, comprising sagittal and axial T1-weighted, T2-weighted, and short tau inversion recovery (STIR) sequences, followed by T1-weighted sequences after intravenous administration of gadolinium contrast. Cervical spine MRI demonstrated increased T2 signal intensity of the C5, C6, and C7 vertebral bodies with milder involvement of C4; these segments show pathological enhancement after contrast administration, in keeping with vertebral osteomyelitis. Pathological tissue was present within the epidural space, intracanalicular, extending from C3 to C7, with high T2 and low T1 signal intensity and enhancement after contrast, reaching a maximum thickness of 5.5 mm. Excess tissue with similar signal and enhancement characteristics occupied the prevertebral space at the C5-C7 level. There was no rim-enhancing collection at either site at this stage and therefore no evidence of an epidural or prevertebral abscess. The spinal cord showed increased T2 signal intensity at the C4-C5 and C6-C7 levels, without contrast enhancement and without cord expansion. The epidural tissue effaced the anterior subarachnoid space but did not compress the spinal cord at any level, and there was accordingly no compressive lesion to account for the myelopathy (Figure 1a).

**Figure 1:**
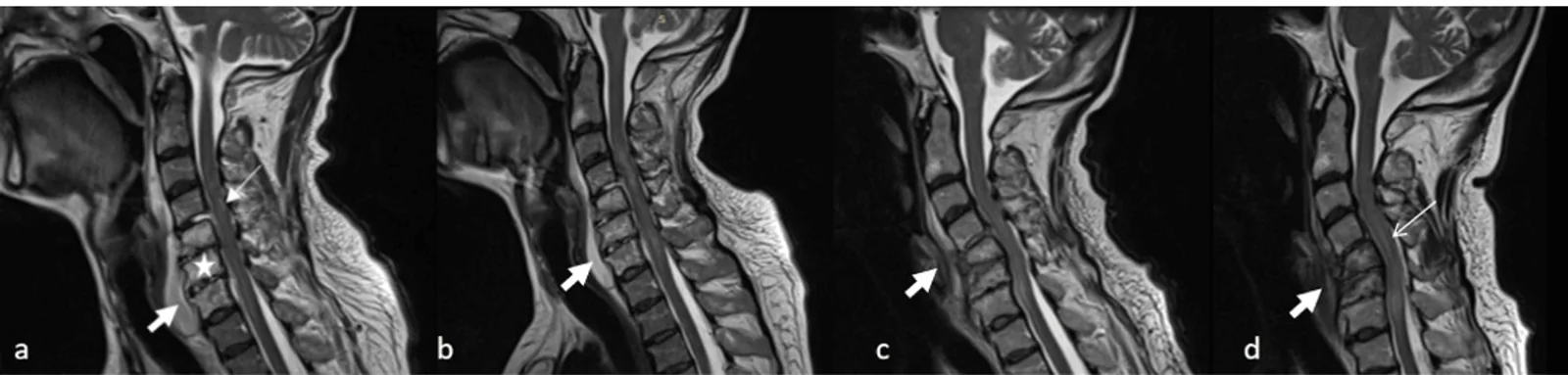
Serial sagittal T2-weighted magnetic resonance imaging of the cervical spine (a) Hospital day 10: Increased T2 signal in the C5-C7 vertebral bodies with milder involvement of C4; intracanalicular epidural pathological tissue extending from C3 to C7 (maximum thickness: 5.5 mm); excess prevertebral tissue at C5-C7; and cord signal abnormality at the C4-C5 and C6-C7 levels. (b) Hospital day 14: The prevertebral tissue has extended to C3-C7 and has assumed cavitary features with a thickened wall, consistent with an organizing abscess cavity. The epidural tissue has decreased in maximum thickness to 4.5 mm, while the cord signal abnormality is relatively more intense than in the previous study. (c) Hospital day 104, shortly before discharge: The prevertebral tissue is notably reduced in extent, the epidural pathological tissue has been reduced, and the vertebral body signal abnormality has become less homogeneous. The cord signal abnormality persists. (d) Approximately three months after discharge: The residual prevertebral tissue has the appearance of an organized cavity with reduced T2 signal, in keeping with fibrotic change rather than persistent fluid collection; the vertebral bodies show areas of low T2 signal consistent with sclerosis and marrow reconstitution. The epidural pathological tissue is significantly reduced, and the cord signal abnormality persists. The thick white arrow indicates the excess prevertebral tissue at the C5-C7 level. The white star in (a) indicates the signal intensity abnormality of the C5, C6, and C7 vertebral bodies, with milder involvement of C4. The thin white arrow in (a) and (d) indicates the signal intensity abnormality of the cervical cord at the C4-C5 and C6-C7 levels, which persists on the final examination. All images shown are T2-weighted; the post-contrast findings referred to in the text are not illustrated.

The patient fulfilled the criteria for ATM [2], and high-dose intravenous methylprednisolone was initiated on hospital day 10. Corticosteroid therapy was continued for 40 days in total, with three attempted dose reductions as described below, and was discontinued on hospital day 49. CSF cultures came back sterile. To investigate alternative or contributing etiologies of disease-associated ATM, a structured workup was undertaken between hospital days 1 and 65. Serum and CSF samples obtained at the time of the initial lumbar puncture were tested for neurotropic viruses by nucleic acid amplification, for blood-borne viral and zoonotic pathogens by serology, and for aquaporin-4 and myelin oligodendrocyte glycoprotein antibodies and cerebrospinal fluid oligoclonal bands. These samples were obtained before the administration of antimicrobial or corticosteroid therapy. Mycobacterial testing, autoimmune serology, and a panel of paraneoplastic and neoplastic markers were performed over the subsequent weeks. All results were negative or within the reference range. The mycobacterial and culture results are listed in Table 3, and the individual tests performed to investigate alternative etiologies, together with the specimen examined and the method used, in Table 4. Structural and systemic mimics were addressed separately by imaging and endoscopic investigation, as described below.

**Table 3:** Microbiological tests CSF: cerebrospinal fluid, MSSA: methicillin-sensitive *Staphylococcus aureus*, MTBC PCR: *Mycobacterium tuberculosis* complex polymerase chain reaction

CSF cultures	Blood cultures	Tuberculosis tests
Negative (four performed in total)	Positive for MSSA (three consecutive sets, hospital days 2, 4, and 6; all subsequent sets negative)	Mantoux test, Ziehl-Neelsen stain, Löwenstein-Jensen culture MTBC PCR, QuantiFERON-TB Gold (all negative)

**Table 4:** Diagnostic workup for alternative etiologies of disease-associated acute transverse myelitis Serological, molecular, and antibody testing were performed on serum and cerebrospinal fluid samples obtained on hospital day 1, except where an alternative day is indicated. Mantoux testing was performed on hospital day 63 (Table 3), and QuantiFERON-TB Gold (Table 3), Brucella serology, and serum immunofixation on hospital day 65. ANA: antinuclear antibodies, anti-HAV: antibodies against hepatitis A virus, anti-HBc: antibodies against hepatitis B core antigen, anti-HBe: antibodies against hepatitis B e antigen, anti-HBs: antibodies against hepatitis B surface antigen, anti-HCV: antibodies against hepatitis C virus, anti-HIV: antibodies against human immunodeficiency virus, AQP4-IgG: aquaporin-4 immunoglobulin G antibodies, β2GPI: beta-2 glycoprotein I, CA 125: cancer antigen 125, CA 19-9: carbohydrate antigen 19-9, C-ANCA: cytoplasmic antineutrophil cytoplasmic antibodies, C3: complement component 3, C4: complement component 4, CSF: cerebrospinal fluid, DNA: deoxyribonucleic acid, ds-DNA: double-stranded DNA, EBV: Epstein-Barr virus, ENA: extractable nuclear antigens, HBeAg: hepatitis B e antigen, HBsAg: hepatitis B surface antigen, HCMV: human cytomegalovirus, HHV-6: human herpesvirus 6, HSV-1: herpes simplex virus type 1, HSV-2: herpes simplex virus type 2, IgG: immunoglobulin G, IgM: immunoglobulin M, MOG-IgG: myelin oligodendrocyte glycoprotein immunoglobulin G antibodies, P-ANCA: perinuclear antineutrophil cytoplasmic antibodies, PCR: polymerase chain reaction, RNA: ribonucleic acid, RNP: ribonucleoprotein, SACE: serum angiotensin-converting enzyme, Sm: Smith antigen, SSA (Ro): Sjögren's syndrome-related antigen A, SSB (La): Sjögren's syndrome-related antigen B, VZV: varicella-zoster virus

Category	Tests performed (specimen; method)	Result
Neurotropic viruses	Measles, rubella, enteroviruses (RNA); HSV-1, HSV-2, VZV, HHV-6, EBV, HCMV (DNA) (serum; PCR)	Not detected
West Nile virus	Antibodies (CSF; immunoassay); RNA (CSF; PCR)	Negative/not detected
Blood-borne viruses	Anti-HIV 1/2, HBsAg, anti-HBs, anti-HBc (total and IgM), HBeAg, anti-HBe; anti-HCV; anti-HAV (total and IgM) (serum; immunoassay)	Negative
Bacterial and zoonotic pathogens	Antibodies against *Listeria monocytogenes* serotypes 1/2a and 4b (serum; immunoassay); Wright agglutination test (brucellosis) (serum; agglutination); Widal test (typhoid fever) (serum; agglutination)	Negative
Demyelinating disease	AQP4-IgG, MOG-IgG (serum; cell-based assay); oligoclonal bands (CSF and serum; isoelectric focusing with immunofixation)	Negative; no CSF-restricted oligoclonal bands (paired CSF and serum)
Systemic autoimmunity	ANA, ds-DNA, ENA (RNP, Sm, SSA/Ro, SSB/La), P-ANCA, C-ANCA, anticardiolipin IgG/IgM, anti-β2GPI IgG/IgM (serum; immunoassay); C3, C4 (serum; nephelometry)	Negative or within reference range
Sarcoidosis	SACE (serum; enzymatic assay)	Within reference range
Neoplastic and paraneoplastic	Serum protein electrophoresis (serum; capillary electrophoresis); immunofixation (serum; immunofixation electrophoresis), tumor markers (CA 125, CA 19-9, alpha-fetoprotein) (serum; immunoassay); immunoglobulins, kappa/lambda light chains, β2-microglobulin (serum; nephelometry); CSF flow cytometry (CSF; microscopy)	Negative; no malignant cells on cytology

The first three blood cultures taken on hospital days 2, 4, and 6 came back positive for methicillin-sensitive *S. aureus* (MSSA) (Table 3). Antimicrobial therapy was adjusted repeatedly over the admission in consultation with infectious diseases. Following the positive blood cultures, empirical ceftriaxone, ampicillin, acyclovir, and vancomycin gave way to metronidazole with cloxacillin; cloxacillin was briefly withdrawn on hospital day 34 because of a generalized rash, and vancomycin was resumed. From hospital day 48, the patient received linezolid and levofloxacin, and from day 56, she received oral moxifloxacin 400 mg once daily. The final regimen, established on hospital day 66, comprised intravenous cloxacillin 750 mg four times daily, oral fusidic acid 500 mg three times daily, and oral moxifloxacin 400 mg once daily; cloxacillin was converted to the oral route at 500 mg four times daily before discharge. Blood cultures became sterile from hospital day 10 onward, and inflammatory markers normalized. The patient started physical therapy sessions.

The CSF pleocytosis resolved by hospital day 15, when the cell count had fallen to within the reference range, although the protein concentration remained elevated on subsequent sampling. Further evaluation and monitoring of the patient was based on repetitive MRI scans. On repeat imaging on hospital day 14, the prevertebral tissue had extended to involve C3-C7 and had assumed cavitary features with a thickened wall, consistent with an organizing abscess cavity measuring 4.5 × 1.1 cm, while the epidural tissue had decreased in maximum thickness to 4.5 mm (Figure 1b). The cavity subsequently reduced to 3.5 × 0.7 cm by hospital day 21 and had resolved by hospital day 34, when the prevertebral tissue no longer showed cavitary characteristics and corresponded to inflammatory tissue alone. Enhancement of the C5-C6 and, to a lesser extent, C6-C7 intervertebral discs appeared for the first time on hospital day 43, indicating progression from vertebral osteomyelitis to spondylodiscitis. On hospital day 62, the vertebral involvement had extended to C4, disc enhancement had intensified, the epidural tissue had increased to 3 mm, and the prevertebral inflammatory tissue had re-extended to a craniocaudal length of 7.5 cm, representing radiological deterioration despite ongoing antimicrobial therapy.

From a clinical point of view, there was a progressive improvement of muscle strength and motility. The patient was experiencing intermittent low-grade fever without other symptoms and with sterile (blood and urine) cultures. To exclude the presence of a different pathogen, not isolated so far, neurosurgical consultation was obtained, and surgical drainage was attempted. However, no abscess was found after surgical exploration of the area, and cultures of the soft tissue in the anterior cervical spinal region and the cervical bone fragment obtained were both sterile. The bone fragment was also sent for biopsy, which revealed inflammatory changes compatible with osteomyelitis. To exclude a different primary site of the infection or another underlying pathology, a whole-body positron emission tomography scan (no focus of infection other than the cervical spine), lower bowel endoscopy (negative for any lesions), CT of the chest and abdomen (negative for a chest or abdominal infection, sarcoidosis, or malignancy), and two transesophageal echocardiograms (negative for endocarditis) were performed at various timepoints throughout the patient's hospitalization, without revealing any pathological features.

Despite the radiological deterioration, the infection came under control, and the patient's neurological status continued to improve. Cervical CT on hospital day 86 demonstrated erosion of the C5 and C6 endplates with loss of vertebral body height and reactive sclerosis. The patient was provided with a cervical collar. Cervical spine MRI obtained on hospital day 104 showed regression of both the prevertebral and the epidural components (Figure 1c). Overall, the patient remained hospitalized for 113 days, under antibiotic therapy, and at the time of discharge, she was able to perform some steps with bilateral support. She was advised to continue the triple antibiotic therapy at home for three more months, perform systematic physiotherapy, and come back afterward for re-evaluation. At follow-up, cervical spine MRI showed persistent increased T2 signal intensity in the C5 and C6 vertebral bodies and at the intervening intervertebral disc, residual pathological tissue in the anterior epidural space at C4-C7, residual inflammatory change in the anterior prevertebral soft tissues at C4-C7, and persistent intramedullary signal abnormality at C4-C5 and C6-C7 (Figure 1d). At that time, the patient was able to walk with bilateral support, and her muscle strength was notably improved. Despite neurological improvement, the presence of persistent inflammation dictated the continuation of antibiotic therapy (cloxacillin and moxifloxacin) for three more months, and at a subsequent time, a new MRI of the cervical spine was suggested. The full chronological sequence of clinical events, investigations, and treatment is summarized in Table 5.

**Table 5:** Chronological summary of the clinical course, investigations, and treatment Day 1 denotes the day of the first presentation. Blank cells indicate that no relevant event was recorded on that day. The apparent neurological deterioration following attempted corticosteroid dose reduction is reported as an observed temporal association; see Discussion. ATM: acute transverse myelitis, CRP: C-reactive protein, CSF: cerebrospinal fluid, CT: computed tomography, FDG-PET: fluorodeoxyglucose positron emission tomography, FIM: Functional Independence Measure, LDH: lactate dehydrogenase, LP: lumbar puncture, MRC: Medical Research Council, MRI: magnetic resonance imaging, MSSA: methicillin-sensitive *Staphylococcus aureus*, WBC: white blood cell count

Day	Clinical events and neurological status	Investigations	Treatment
1	Presentation after 10 days of generalized weakness, gait deterioration, neck pain, and sore throat; temperature: 38.2°C, nuchal rigidity, generalized weakness	Brain CT normal; cervical CT no acute compressive lesion; LP: 130 cells/μL (88% lymphocytes), glucose: 29 mg/dL, protein: 885 mg/dL, LDH: 76 U/L, CRP: 30.5 mg/dL, WBC: 36.2 K/μL	Empirical ceftriaxone, ampicillin, vancomycin, and acyclovir
2	Progressive deterioration to flaccid quadriparesis with a bilateral C4 sensory level; FIM motor 13, cognitive 30	Blood culture set 1: MSSA	-
4	Formal neurological examination: alert, preserved higher cortical function, normal oculomotor responses, intact cranial nerves; MRC grade: right upper limb: 4/5 proximal, 3/5 distal; left upper limb: 5-/5 proximal, 3/5 distal; lower limbs: 2+/5 bilaterally; deep tendon reflexes abolished in all four limbs; plantar responses absent; Barré and Mingazzini signs positive bilaterally; Hoffmann sign negative; cerebellar testing unremarkable; unable to maintain a standing position	Blood culture set 2: MSSA; abdominal CT unremarkable	-
5	-	Chest CT: bilateral pleural effusions; retropharyngeal soft tissue 3-4 cm extending to the T1 level	-
6	-	Blood culture set 3: MSSA; repeat LP: 160 cells/μL (84% lymphocytes), glucose: 48 mg/dL, protein: 844 mg/dL	-
7	-	Electrodiagnostic testing	-
10	Diagnostic criteria for ATM fulfilled	Blood cultures sterile (and all subsequent sets); cervical MRI: C5-C7 vertebral osteomyelitis (C4 milder), epidural pathological tissue C3-C7 (maximum thickness 5.5 mm), prevertebral inflammatory tissue C5-C7, cord T2 hyperintensity at C4-C5 and C6-C7 without enhancement (Figure 1a)	High-dose intravenous methylprednisolone initiated
14	Physiotherapy commenced	Cervical MRI: epidural tissue 4.5 mm; prevertebral collection develops abscess characteristics, craniocaudal extent 8.2 cm, cavity 4.5 × 1.1 cm; cord signal relatively intensified (Figure 1b)	-
15	-	LP: 10 cells/μL, protein: 125 mg/dL; pleocytosis resolved	-
21	-	Cervical MRI: epidural tissue 2.5 mm; prevertebral craniocaudal extent 7.3 cm; cavity 3.5 × 0.7 cm	-
25	-	-	Initial antimicrobial regimen stopped; metronidazole and cloxacillin started
29	-	First transesophageal echocardiogram: no vegetations	-
34	Generalized rash	Cervical MRI: abscess cavity resolved; prevertebral extent 4.7 cm; epidural tissue 2 mm, C4-C7; new posterior paraspinal soft tissue edema	Cloxacillin withdrawn; vancomycin resumed
43	-	Cervical MRI: new intervertebral disc enhancement at C5-C6 and C6-C7, indicating evolution to spondylodiscitis	-
48	-	-	Vancomycin stopped; linezolid and levofloxacin started
49	-	-	Corticosteroids discontinued after 40 days; three attempted dose reductions during the first month had each been followed by apparent neurological deterioration; no deterioration followed final withdrawal
56	-	-	Oral moxifloxacin 400 mg once daily
62	-	Cervical MRI: imaging deterioration; new C4 involvement, disc enhancement intensified, epidural tissue 3 mm, prevertebral extent 7.5 cm	-
63	-	LP: 4 cells/μL, protein: 383 mg/dL; Mantoux test negative	-
66	Multidisciplinary infectious diseases consultation	Second transesophageal echocardiogram: no vegetations	Final regimen: intravenous cloxacillin 750 mg x 4, oral fusidic acid 500 mg three times daily, oral moxifloxacin 400 mg once daily
70	-	Lower gastrointestinal endoscopy: no lesions	-
72	Surgical exploration of the anterior cervical region: no abscess found	Prevertebral soft tissue and cervical bone cultures negative; bone histology consistent with osteomyelitis, no evidence of malignancy	-
86	-	Cervical CT: C5-C6 endplate erosion, vertebral height loss, sclerosis	Rigid cervical collar; no neurosurgical intervention indicated
100	-	Whole-body FDG-PET: no infectious focus identified	-
104	-	Cervical MRI: regression of both the prevertebral and the epidural components (Figure 1c)	-
113	Discharge; improved motor function; able to take some steps with bilateral support; FIM: motor, 43; cognitive, 33	No evidence of active infection; inflammatory markers and procalcitonin within reference range	Oral cloxacillin, fusidic acid, and moxifloxacin continued for three further months
201	Follow-up assessment; walking with bilateral support; muscle strength notably improved	Cervical MRI: persistent cord T2 hyperintensity at C4-C5 and C6-C7, significantly reduced epidural pathological tissue at C4-C7, significantly reduced prevertebral inflammation at C4-C7 (Figure 1d)	Cloxacillin and moxifloxacin continued for three further months

## Discussion

We herein describe a rare case of cervical ATM associated with *S. aureus* bacteremia and vertebral osteomyelitis progressing to spondylodiscitis. Various infectious agents have been associated with myelitis, notably herpesviruses, paramyxoviruses, picornaviruses, flaviviruses, orthomyxoviruses, *Treponema pallidum* (syphilis), *Mycobacterium tuberculosis*, *Borrelia burgdorferi* (Lyme disease), *Actinomyces*, *Coccidioides immitis*, *Aspergillus*, *Blastomyces dermatitidis*, *Taenia solium* (neurocysticercosis), *Schistosoma*, *Gnathostoma*, and *Angiostrongylus* [3]. However, to our knowledge, only a limited number of cases have been published so far describing ATM associated with *S. aureus* infection. Karakonstantis et al. reviewed six previously reported cases of ATM etiologically related to *S. aureus* infection with sufficient clinical detail for comparison [6-11] and described a seventh case of their own [12]. Another two cases have been recently published by Reffo et al. [13] and Vikhe et al. [14]. All cases (including the present case, 10 in total) are summarized in Table 6 and Table 7. Experience gained from these reports is important to ensure timely diagnosis and optimal management of this rare clinical entity, considering the significant morbidity and mortality in such cases.

**Table 6:** Reported cases of acute transverse myelitis associated with Staphylococcus aureus infection: clinical, microbiological, and imaging features Case identification: Cases were identified through a full-text search of Google Scholar and a title/abstract search of PubMed, using the terms "acute transverse myelitis" and "*Staphylococcus aureus*", conducted in September 2026 without date restriction and limited to reports published in English. The search was non-systematic and unregistered; no protocol, duplicate screening, or formal risk-of-bias assessment was applied, and the table is intended as a narrative comparison rather than a systematic review. Data extraction: Data were extracted from the full text of each source publication. Where a value could not be established from the source, the cell reads "not reported"; where an investigation was explicitly not performed, this is stated. Note on attribution: Vikhe et al. attributed the myelitis primarily to concurrent hepatitis C virus infection, with MSSA endocarditis as a contributing factor [14]. In Friess and Wasenko, no biopsy was performed, and the diagnosis was presumptive [11]. In Kalkan et al. [10] and Friess and Wasenko [11], pre-existing neurological conditions (meningomyelocele with ventriculoperitoneal shunt; paraplegia following trauma) limit interpretation of the reported neurological findings. ADA: adenosine deaminase, CA-MRSA: community-acquired methicillin-resistant *Staphylococcus aureus*, CSF: cerebrospinal fluid, CT: computed tomography, FDG-PET: fluorodeoxyglucose positron emission tomography, HCV: hepatitis C virus, LDH: lactate dehydrogenase, MIC: minimum inhibitory concentration, MRI: magnetic resonance imaging, MRSA: methicillin-resistant *Staphylococcus aureus*, MSSA: methicillin-sensitive *Staphylococcus aureus*, PCR: polymerase chain reaction, PVL: Panton-Valentine leucocidin, RT-PCR: reverse transcription polymerase chain reaction, SCCmec: staphylococcal cassette chromosome mec, ST: sequence type, TEE: transesophageal echocardiography

Study (year, country)	Age/sex	Organism and positive specimens	Extraspinal or adjacent focus	Cord lesion	Vertebral/paravertebral involvement	CSF
Present case	56/female	MSSA, 3 consecutive blood culture sets (hospital days 2-6); CSF culture and PCR negative; prevertebral soft tissue and bone cultures negative	None identified outside the cervical spine (PET, TEE, chest/abdominal CT, endoscopy negative)	C4-C5 and C6-C7 (discontinuous)	C5-C7 osteomyelitis (C4 milder), progressing to spondylodiscitis; anterior prevertebral inflammation C5-C7; anterior epidural tissue C3-C7; bone biopsy consistent with osteomyelitis	130/μL (88% lymphocytes), glucose: 29 mg/dL (CSF:serum: 0.40), protein: 885 mg/dL, LDH: 76 U/L
Eduwu et al. (2017, USA) [6]	26/female, previously healthy	MRSA, blood, and sputum cultures; persistently positive blood cultures on hospital days 2, 4, 6, and 18 despite therapeutic vancomycin troughs; vancomycin MIC 2 μg/mL by Etest; CSF culture negative (day 4, on vancomycin)	Necrotizing pneumonia with bilateral cavitary lesions; transthoracic and three transesophageal echocardiograms negative for vegetation or intracardiac abscess	C3-T2, diffuse myelitis; no cord compression or abscess	C5-T1 osteomyelitis with phlegmon extending C3-T2	58/μL (81% neutrophils), glucose: 55 mg/dL (serum: 136; CSF:serum: 0.40), protein: 537 mg/dL
He et al. (2016, China) [7]	34/male	MRSA, pus, blood, and CSF cultures	Left psoas abscess (>300 mL), following paravertebral injection	C2-C3, with cord expansion	Abscess invaded the lumbar intervertebral space; lumbar vertebral histology showed non-specific inflammation; no cervical vertebral involvement	5000/μL, glucose: 11 mg/dL, protein: 500 mg/dL, opening pressure: 180 mmH₂O
Kulkarni et al. (2009, India) [8]	19/male	CA-MRSA, blood, and abscess cultures; PVL-positive, SCCmec type V, ST772	Bronchopneumonia; cervicothoracic paraspinal pyomyositis with abscess (~1 L pus)	C2-C4, with diffuse cervical meningeal enhancement	T1 vertebral osteomyelitis	470/μL (95% neutrophils), glucose: 55 mg/dL, protein: 400 mg/dL; Gram stain and culture negative
Saini et al. (2014, Singapore) [9]	79/female	MSSA, blood, and CSF cultures	None identified	T11, non-enhancing	None; an enhancing L1 vertebral lesion was unchanged from imaging one year earlier; T10/11 scintigraphic uptake without MRI correlate, judged non-specific	813/μL (95% neutrophils), glucose: 76 mg/dL (CSF:serum: 0.70), protein: 280 mg/dL; oligoclonal bands negative
Kalkan et al. (2012, Turkey) [10]	13/female	MRSA, bronchoalveolar lavage	Ventilator-associated pneumonia; prior meningomyelocele repair and ventriculoperitoneal shunt	Diffuse, anterior cord hyperintensity	None reported	Not reported
Friess and Wasenko (1997, USA) [11]	41/female	*Staphylococcus aureus*, blood culture (methicillin susceptibility not reported)	Multiple ring-enhancing cerebral lesions; thickened mitral valve suggesting endocarditis; paraplegic since a road traffic accident 3 years earlier	C2-C7, homogeneously enhancing, central and right-sided; no cord expansion	None reported	Not obtained
Karakonstantis et al. (2017, Greece) [12]	79/male	MSSA, 2 blood cultures; CSF culture (two lumbar punctures) and PCR negative, on cefuroxime for 2 days; paravertebral tissue culture negative (day 14)	None identified (TEE, abdominal ultrasound, chest CT negative)	C3-C5, swollen and enhancing	C5-T1 osteomyelitis; prevertebral phlegmon; bone biopsy consistent with osteomyelitis	298/μL (58% lymphocytes, 35% neutrophils), glucose: 71 mg/dL (CSF:serum: 0.44), protein: 1738 mg/dL
Reffo et al. (2025, Italy) [13]	60/male	MSSA, 3 blood cultures: CSF culture negative; CSF broad-range multiplex RT-PCR positive for *S. aureus*	None identified (TEE, brain MRI, whole-body FDG-PET negative)	C5 and T1, right-sided, enhancing	Explicitly excluded on MRI and PET	566/μL, glucose: 69 mg/dL (CSF:serum: 0.80), protein: 330 mg/dL, lactate: 4 mmol/L
Vikhe et al. (2024, India) [14]	29/female	MSSA, blood culture; concurrent HCV viremia (>6.4 million/dL)	Tricuspid valve endocarditis (vegetation: 1.27 × 1.32 cm)	T6-T9, enhancing	None reported	32/μL (90% lymphocytes), glucose: 52 mg/dL, protein: 72 mg/dL, ADA: 4 U/L

**Table 7:** Reported cases: treatment, outcome, and follow-up Follow-up is the interval from presentation to the last documented neurological assessment. The considerable variation in follow-up duration, completeness of neurological documentation, and use of standardized outcome measures across these reports limits direct comparison between cases, and outcomes are therefore reported as the endpoint described by each source rather than assigned to a common grading scale. FIM: Functional Independence Measure, IgA: immunoglobulin A, IgM: immunoglobulin M, IV: intravenous, IVIG: intravenous immunoglobulin, MRI: magnetic resonance imaging, MRSA: methicillin-resistant *Staphylococcus aureus*, MU: megaunits

Study	Antimicrobial therapy	Corticosteroid/immunomodulatory therapy	Surgery	Neurological status at last assessment	Follow-up
Present case	Empirical ceftriaxone, vancomycin, ampicillin, acyclovir; switched to per os fusidic acid 500 mg × 3, IV cloxacillin 750 mg × 4, and per os moxifloxacin 400 mg × 1; fusidic acid withdrawn beyond 6 months	High-dose IV methylprednisolone started at day 10 and lasted until day 49	Cervical exploration; no abscess found	Walking with bilateral support; FIM motor 13→43, cognitive 30→33	6.5 months
Eduwu et al. [6]	Empirical vancomycin + levofloxacin; switched to daptomycin 10 mg/kg daily + linezolid 600 mg 12-hourly IV for persistent bacteremia; gentamicin 1 mg/kg 8-hourly added day 18, stopped at 2 weeks; linezolid stopped at 4 weeks; daptomycin continued 8 weeks from first negative culture	None reported	None; neurosurgery advised conservative management	Symptom-free with no neurological sequelae; complete radiological resolution of phlegmon and cord inflammation on end-of-treatment MRI	8 weeks after completion of antimicrobial therapy
He et al. [7]	Vancomycin 10 days, then oral linezolid 6 weeks	Not reported	Psoas abscess drainage	Upper limb power 3/5, moving fingers and hands, sensation above T4, weaned from ventilator by day; remained ventilator-dependent overnight	6 months
Kulkarni et al. [8]	Empirical ampicillin, amikacin, ceftriaxone, metronidazole; changed to vancomycin for 4 weeks	Not reported	Drainage of paraspinal abscess	No neurological deficit; asymptomatic	2 months after a 6-week admission
Saini et al. [9]	IV ceftriaxone 1 g daily, then IV crystalline penicillin 4 MU 4-hourly for 6 weeks	IV methylprednisolone, discontinued once cultures returned positive	None	No improvement; then lost to follow-up	6 weeks
Kalkan et al. [10]	Linezolid (for MRSA pneumonia)	Deferred because of active infection	None	Improved with physical therapy	Not reported
Friess and Wasenko [11]	Gentamicin + oxacillin	Not reported	None	Right upper limb function returned to baseline (pre-existing paraplegia unchanged); cord and cerebral lesions reduced on repeat MRI	2 weeks
Karakonstantis et al. [12]	Empirical dexamethasone, ceftriaxone, vancomycin, ampicillin, acyclovir; IV cloxacillin 2 g 4-hourly from day 4 for 6 weeks	Dexamethasone, then high-dose IV methylprednisolone 1 g daily; no response	Exploration at day 14; no abscess found	Remained tetraplegic; died some months later	2 months
Reffo et al. [13]	Ampicillin, ceftriaxone, levofloxacin; daptomycin + ceftaroline + fosfomycin; oxacillin + levofloxacin + rifampin; oral rifampin + levofloxacin; 4 weeks total	Dexamethasone 0.15 mg/kg/day; IgA/IgM-enriched immunoglobulin	None	Walking with minimal aids	3 months
Vikhe et al. [14]	Vancomycin 1 g IV twice daily + ceftriaxone 2 g IV daily, 6 weeks; sofosbuvir-velpatasvir 40/10 mg daily, 3 months	Methylprednisolone 1 g IV daily × 5 days (minimal response); IVIG 2 g/kg over 5 days	None	Resolution of urinary retention, partial motor recovery	"Several weeks", not further specified

Our patient presented with signs and symptoms indicating systemic infection (fever and markedly elevated inflammatory blood markers) and CNS insult, possibly localized in the spinal cord (lack of encephalopathy, severe neck pain, nuchal rigidity, flaccid quadriparesis, and sensory dysfunction). Initial steps in the emergency paraclinical evaluation were (i) obtaining blood cultures, (ii) performing brain and spinal CT to exclude a compressive lesion of the spinal cord, and consequently, (iii) proceeding to lumbar puncture for CSF analysis. CSF results indicated possible infection (pleocytosis, elevated protein concentration, and low glucose levels) but were otherwise inconclusive. The aforementioned clinical presentation raised the suspicion of an ongoing infectious spinal syndrome, but several other causes of myelitis could not be definitively excluded at that time.

High-dose intravenous methylprednisolone was initiated once the patient fulfilled the criteria for ATM [2], under established antimicrobial cover. Although corticosteroids in the setting of documented bacteremia are counterintuitive, the mechanism of the cord injury was at that point unestablished, and immune-mediated ATM (for which corticosteroids are the accepted first-line therapy despite the absence of randomized evidence [15]) remained within the differential. The rationale parallels adjunctive corticosteroid use in bacterial CNS infection: attenuation of the inflammatory response and of vasogenic edema within the non-compliant spinal canal, where cord swelling may cause secondary ischemic injury and irreversible deficit. The principal risk of this approach was impaired bacterial clearance with extension of the infection, and whether prolonged corticosteroid exposure contributed to the radiological deterioration observed on hospital day 62 cannot be determined.

After blood cultures turned positive for *S. aureus*, the antibiotic treatment was modified accordingly, the infection was subsequently brought under control, and the patient's neurological state improved over the following months. It is important to note that the antibiogram for the *S. aureus* isolated showed high susceptibility of the pathogen to the empirical treatment for meningoencephalitis that was initially administered; nevertheless, we chose to switch to antibiotics combining narrow- and wide-spectrum effects against Gram-positive bacteria, especially MSSA, and a good bone penetration profile. It is worth noting that cloxacillin alone has poor lipid solubility, and its CSF penetration may be insufficient. It is also worth noting that fusidic acid was withdrawn in the longer term; beyond six months, we challenged its therapeutic utility, which includes disrupting bacterial biofilms and rapidly reducing bacterial load to control the infection at an early stage, to be outweighed by the potential harms to the patient of receiving multiple antibiotic regimens for such a prolonged period.

In our case, the mechanism of the spinal cord damage remains unclear. It is debatable whether the myelitis was the result of an intramedullary spread of the microbial agent, directly/contiguously by the adjacent infected tissues, or the result of a reactive, immune-mediated (parainfectious) process. In support of the first hypothesis are (i) the isolation of methicillin-sensitive *S. aureus* from three consecutive sets of blood cultures obtained over the first six hospital days, indicating sustained rather than transient bacteremia, (ii) hypoglycorrhachia, and (iii) moderately increased CSF protein, suggestive of bacterial CNS infection. It should be noted that these CSF findings are not specific: hypoglycorrhachia is also seen in meningeal carcinomatosis, while an elevated protein concentration may result from a spinal block due to compressive lesions (tumor, abscess, and herniated disc), meningeal carcinomatosis, or* Mycobacterium tuberculosis* infection. In our case, all these scenarios were excluded. Moreover, (iv) vertebral infection confirmed by imaging and histology was present immediately adjacent to the affected cord segments, and the anatomical contiguity of the two processes is consistent with direct spread.

On the other hand, we should note that (i) *S. aureus* was never isolated in CSF or cultures of the tissue biopsies and bone fragment, which, however, were taken several weeks after initiation of treatment with antibiotics. Second, (ii) evidence that does not quite match the first hypothesis is the concentration of CSF LDH. The latter has been reported to rise significantly in CNS bacterial infections, contrary to viral or noninfectious causes [16]. In our case, LDH levels were only slightly increased (76 U/L; the normal range is <40 U/L for adults). A further observation, of uncertain significance, is that three attempts to reduce the corticosteroid dose during the first month of hospitalization were each followed by apparent neurological deterioration, which improved again when the dose was increased. We report this as an observed temporal association only. It cannot be interpreted as evidence of an immune-mediated mechanism: the observations were not blinded or protocolized, antimicrobial therapy was being adjusted over the same interval, corticosteroid withdrawal can produce non-specific systemic symptoms that may be mistaken for neurological decline, and the natural fluctuation of an evolving cord lesion offers an equally plausible explanation. Corticosteroids were ultimately withdrawn on hospital day 49, after 40 days of treatment, without recurrence of the deterioration seen on the earlier reduction attempts. A further consideration is that our patient had a background of hypothyroidism and vitiligo, a combination suggesting an underlying predisposition to autoimmunity. Whether such a predisposition increases susceptibility to a parainfectious immune-mediated myelitis is not established, and we note it as a feature of the case rather than as evidence for the second hypothesis. In cases of parainfectious ATM, spinal cord damage can be caused by antigen-mediated disease or mechanisms of molecular mimicry [17]. Regarding antigen-mediated disease, staphylococcal enterotoxins have been described to excessively stimulate the immune system, triggering autoimmune responses [18].

The neurological deficit in this patient was attributable to the cord lesion rather than to the adjacent structural pathology: the flaccid quadriparesis, generalized areflexia, and sensory level corresponded to the intramedullary signal abnormality at C4-C5 and C6-C7, whereas the epidural tissue effaced the anterior subarachnoid space without compressing the cord, and the vertebral and prevertebral changes accounted for the neck pain and the systemic inflammatory response, but not for the myelopathy. Notably, the intramedullary abnormality was discontinuous, involving C4-C5 and C6-C7 with intervening normal cord, a distribution that does not correspond to the continuous C3-C7 extent of the adjacent epidural and vertebral disease. This discordance between the anatomy of the cord lesion and that of the adjacent infection constitutes a further argument against direct contiguous invasion as the sole mechanism.

The distinction between an inflammatory and vascular origin of the underlying condition was not obvious; therefore, a full workup was conducted to define any structural, metabolic, and inflammatory markers that would facilitate the diagnosis. Compressive myelopathy was excluded by MRI, while spinal cord infarction would have a more acute presentation and a vascular distribution of neurological findings on clinical examination (anterior spinal artery syndrome). Systemic diseases associated with ATM were excluded serologically (Table 4). A demyelinating etiology was considered unlikely based on the clinical presentation and MRI appearances and was further argued against by negative serum AQP4-IgG and MOG-IgG antibodies and the absence of oligoclonal bands in the CSF. Finally, the response of the patient to steroid infusions and evidence of inflammation in the CSF made the diagnosis of venous congestion less likely [19].

Following modification of antimicrobial therapy, the patient's neurological status improved progressively over the subsequent months. Because targeted antimicrobial treatment, corticosteroid therapy, surgical exploration, and an intensive rehabilitation program were delivered over an overlapping period, the contribution of any individual intervention to this recovery cannot be determined, and the improvement is best described as the observed clinical course rather than as the effect of a particular treatment (Table 5). This improvement was reflected in the Functional Independence Measure (FIM) [20], assessed on hospital days 2 and 113. The motor subscale score rose from 13 to 43 of 91, and the cognitive subscale score from 30 to 33 of 35. No further FIM assessment was performed at the follow-up visit, so the extent of recovery beyond discharge is not quantified. Findings consistent with control of the infection were (i) sterilization of blood cultures by hospital day 10 and (ii) resolution of fever and normalization of inflammatory markers. Lastly, it is worth noting that the neurological improvement of our patient is in accordance with the outcome in previously described younger patients, whereas older patients seem to have poorer clinical trajectories (Table 6 and Table 7).

This report has several limitations. As a single case, it can neither establish causation nor support inferences about the relative merits of any treatment strategy. The mechanism of cord injury was not established: *S. aureus* was not recovered from CSF, cord tissue, or adjacent structures, and the specimens that might have resolved this were obtained only after several weeks of antimicrobial therapy. Broad-range molecular testing of CSF, which has been reported to identify *S. aureus* in a comparable case [13], was not performed. Antimicrobial therapy, corticosteroids, surgical exploration, and rehabilitation were delivered concurrently, so their individual contributions to the observed recovery cannot be separated. Finally, the neurological examination at presentation was not documented using the International Standards for Neurological Classification of Spinal Cord Injury, which limits direct comparison with previously reported cases.

## Conclusions

We describe a rare association of acute transverse myelitis with cervical spondylodiscitis and MSSA bacteremia. Only a small number of comparable cases have been reported, and the present case illustrates the diagnostic difficulty that arises when myelitis occurs alongside adjacent vertebral and epidural infection, since direct infectious involvement of the cord and a parainfectious immune-mediated process cannot readily be distinguished on clinical, laboratory, or radiological grounds. Both mechanisms remain plausible here, and neither was established. What this case does support is a narrower point: in a patient presenting with acute myelopathy and features of systemic infection, blood cultures and dedicated spinal imaging should be obtained early, because an occult adjacent focus of infection may be present and requires treatment in its own right irrespective of how the cord injury is ultimately explained. The favorable neurological trajectory in this patient occurred during concurrent antimicrobial, corticosteroid, surgical, and rehabilitative management and cannot be attributed to any single component of that regimen.
